# Ordered array of Ag semishells on different diameter monolayer polystyrene colloidal crystals: An ultrasensitive and reproducible SERS substrate

**DOI:** 10.1038/srep32314

**Published:** 2016-09-02

**Authors:** Zao Yi, Gao Niu, Jiangshan Luo, Xiaoli Kang, Weitang Yao, Weibin Zhang, Yougen Yi, Yong Yi, Xin Ye, Tao Duan, Yongjian Tang

**Affiliations:** 1 Joint Laboratory for Extreme Conditions Matter Properties, Southwest University of Science and Technology, Mianyang 621900, China; 2 Co-Innovation Center for Energetic Materials, Southwest University of Science and Technology, Mianyang 621900, China; 3 Research Center of Laser Fusion, China Academy of Engineering Physics, Mianyang 621900, China; 4 Department of Physics, Dongguk University, Seoul, 100715, Korea; 5 College of Physics and Electronics, Central South University, Changsha 410083, China

## Abstract

Ag semishells (AgSS) ordered arrays for surface-enhanced Raman scattering (SERS) spectroscopy have been prepared by depositing Ag film onto polystyrene colloidal particle (PSCP) monolayer templates array. The diversified activity for SERS activity with the ordered AgSS arrays mainly depends on the PSCP diameter and Ag film thickness. The high SERS sensitivity and reproducibility are proved by the detection of rhodamine 6G (R6G) and 4-aminothiophenol (4-ATP) molecules. The prominent enhancements of SERS are mainly from the “V”-shaped or “U”-shaped nanogaps on AgSS, which are experimentally and theoretically investigated. The higher SERS activity, stability and reproducibility make the ordered AgSS a promising choice for practical SERS low concentration detection applications.

Since the surface enhanced Raman signals have been found on the surface of metal nanostructure, the surface enhanced Raman scattering (SERS) technique has been used as a powerful analytical tool in widely applications, including measurements, chemical, and biological sensing^1^,^2^,^3^. There are two main mechanisms at work for SERS: the chemical (CM) mechanism and the electromagnetic (EM) mechanism. The EM mechanism is recognized as the largest contribution^4^. Generally speaking, the EM mechanism is owing to the enhanced local electric field that results in a significant increase in the cross section of the Raman scattering^5^,^6^. The enormous enhancement of SERS is mainly derived from the “hot spots” on the metal surfaces^7^,^8^.

In order to realize satisfactory reliability and sensitivity of SERS substrates, the homogeneous Raman signals and reproducible preparation are very important. Lots of SERS substrates with “hot spots” have been prepared by different ways including chemical functional method^9^, electron lithography technique^10^ and self-assembled aggregation^11^,^12^. Relatively speaking, as a simple, economic and effective way, nanosphere lithography (NSL) can be used to fabricate uniformly ordered nanoparticle arrays because of its good monodispersity, wide size range and realizable^13^,^14^,^15^,^16^,^17^,^18^,^19^,^20^,^21^,^22^,^23^,^24^,^25^,^26^. For instance, by using NSL, Van Duyne’s group fabricated Au and Ag nanoparticle array on self-assembly monolayer polystyrene or silica nanospheres^13^,^14^,^15^,^16^. Sugawa’s group prepared periodic Au half-shells and ordered arrays of copper nanostructures on the upper hemispheres of two-dimensional silica^17^,^18^. Cai’s group fabricated ordered Ni hollow sphere arrays and Ag nanoshell arrays on a self-assembled polystyrene monolayer via electrodeposition process^19^,^20^, and they fabricated Ag/Au bimetallic shell composite microsphere arrays and Au opening-nanoshell ordered arrays on a self-assembled polystyrene monolayer via sputtering deposition^21^,^22^. Zhang *et al*. fabricated SiO_2_-Ag nanocap arrays via thermal evaporation deposition on two-dimensional polystyrene spheres (PS) colloidal particle templates^23^. Zhao *et al*. fabricated Ag semishell arrays with controlled size and gaps by combining magnetron sputtering and reactive ion etching^24^. As SERS substrates, these ordered arrays have showed large area uniform Raman activity^25^,^26^.

In order to produce monolayer polystyrene colloidal particle (PSCP) templates array with a higher order, the predominant techniques were based on Langmuir-Blodgett processes^27^. However, the procedure was time consuming and required special equipment. The pH value of the water solutions was adjusted to tune the balance between electrostatic repulsion and van der Waals attraction as well as capillary forces for large areas highly ordered monolayers colloidal crystals^28^. However, there would be residual impurities on the substrate because of addition of mineral acid for adjusting the PH value. Surfactants like sodium dodecyl sulfonate (SDS) also are added to colloidal suspensions in advance for facilitating the dispersion of PS microspheres on surface initial substrate. Yet, it has little effect for second selfassembly on air-water interface^29^.

In this paper, a SERS substrate was developed based on the colloidal self-assembly of PSCP modulated by magnetron sputtering Ag coating film thickness control. The self-assembly process of the PSCP, regulation of the ordered arrays of Ag semishells (AgSS), mechanism for the formation of SERS-active structures, and characterization of the SERS substrates performance were explored to study and evaluate the developed SERS substrates. The morphologies and the SERS properties of the AgSS were modified by adjusting the PSCP diameter and the Ag film thickness. This research can provide an effective strategy for preparing highly sensitive and reproducible SERS substrates, which can give a perfect way for other complex nanostructures as highly active SERS substrates.

## Result and Discussion

The high order monolayer PSCP films depend on many experiment parameters. Some parameters have been proven to be the most significant parameters, including the substrate wetting, solution concentration, velocity (spin speed) and spin acceleration^30^,^31^. Many previous research papers have reported the relationship between spin coating parameters, the coverage and ordering. Herein, we built a system research that certain size of PSCP ranging from 160 to 1300 nm. On this multi parameter problem, it is apparent that careful optimization for each parameter to obtain optimal SERS substrate quality. As we know that cluster formation is enhanced with increasing microsphere concentration and change of the particle surface polarity takes place during the spinning coating and drying. In our experiment, the important case involves self-assembly a high ordered monolayer on the air/water interface and transforming it to the substrate. After the substrate with PSCP was immersed in into DI water slowly, the PSCP float on the surface of water. However, the PSCP exhibited disorder because they didn’t self-assemble on surface of water. If the water surface tension was changed at the side of colloidal film, the colloidal film with many PSCP on surface contract a high ordered monolayer colloidal crystals. The water surface tension can be changed by surfactant (SDS). Herein, we have systematically investigated some factors influencing the monolayer quality that include the concentration of surfactant (SDS), concentration of microsphere and rotation speed, as shown the Fig. S1–3. Figure S1 show SEM images of PS spheres monolayer colloidal crystals fabricated at different concentration of PS spheres. The volume of SDS (0.01 mol/L) is 800 μl, and the spin speed is 2000 rad/min. The diameter of sphere is 300 nm. These images indicate that different layer quality can be fabricated at different concentration. On the basis of experimental results, the optimal condition for the formation of the order monolayer PSCP films is that the concentration of PS spheres is 3 wt%. Figure S2 show SEM images of PS spheres (300 nm) monolayer colloidal crystals fabricated at different volume of SDS (0.01 mol/L). The concentration of PS is 3 wt%, and the spin speed is 2000 rad/min. On the basis of the Fig. S2, the optimal condition for the formation of the order monolayer PSCP films is that the volume of SDS (0.01 mol/L) is 800 μl. Figure S3 show SEM images of PS spheres (300 nm) monolayer colloidal crystals fabricated at different spin speed. The concentration of PS is 3 wt%, and the volume of SDS (0.01 mol/L) is 800 μl. As shown the Fig. S3, the optimal condition for the formation of the order monolayer PSCP films is that the spin speed is 2000 rad/min. To give an instance, six diameters (160, 300, 430, 600, 820 and 1300 nm) of PSCP were chosen and a series of experiments adjusting the ordering degree of PSCP monolayer arrays, as shown the Table 1.

**Figure 1:**
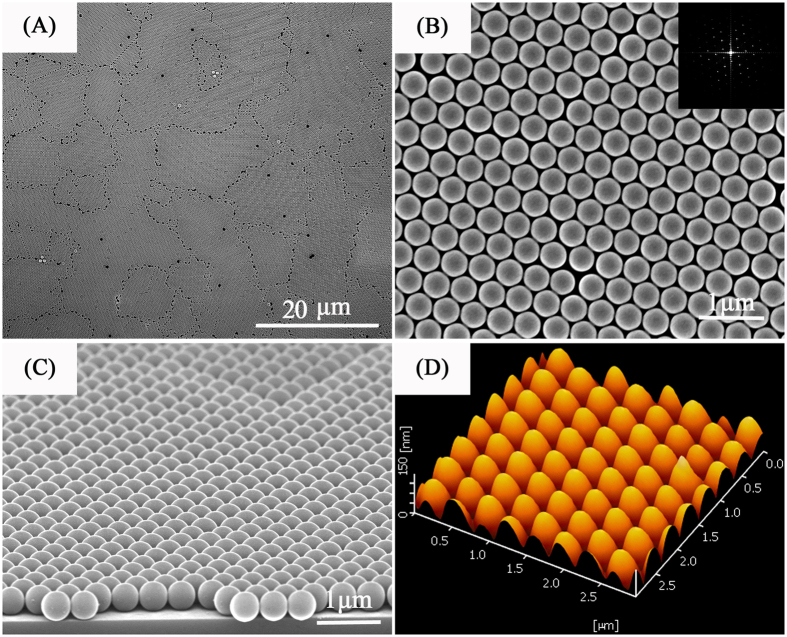
The images of the monolayer PSCP array films (300 nm): (**A**–**C**) SEM images, (**D**) AFM. The inset in panel (B) shows a fast Fourier transform (FFT) pattern.

Figure 1 displays the typical SEM images and AFM image of the monolayer PSCP array films (300 nm). The typical monolayer PSCP arrays (300 nm) were prepared as following: The concentration of PSCP is 3 wt%, the volume of SDS (0.01 mol/L) is 800 μl and spin speed is 2000 rad/min. It can be clearly observed from Fig. 1A that the large coverage and uniform monolayer area reaching more than 0.4 cm^2^ has been obtained. However, the uniform monolayer areas of PSCP array film obtained for the present experimental condition are not always defect free. The PSCP were arranged in a long-range order with few defects, which have little influence if these substrates are applied in many fields, such as for SERS^25^. In addition, the SEM image with higher magnification in Fig. 1B displays clear evidence that the monolayer exhibits a close packed hexagonal ordered arrangement of PSCP. The sharp peaks in Fourier transform of low magnification images (inserts in Fig. 1B) prove the presence of long range order PSCP arrays with a hexagonal symmetry. The side-view FESEM image in the Fig. 1C further demonstrates the monolayer characterization of this film. Figure 1D shows the AFM image of monolayer PSCP array film in an area of 3 μm × 3 μm. The images shows that the monolayer PSCP arrays exhibit hexagonal packing on the substrate over a large area, which contribute to the formation of high-quality ordered array of PSCP.

**Figure 2:**
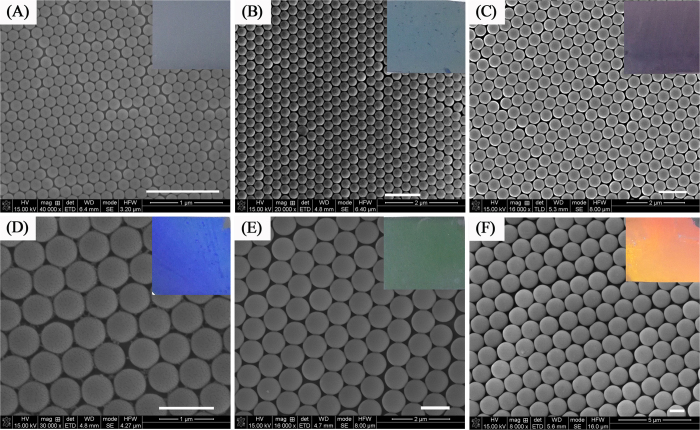
Top-view SEM images for monolayer PS colloidal-crystal films prepared using PS microspheres with different diameter (scale bar 1 μm): (**A**)160 nm; (**B**) 300 nm; (**C**) 430 nm; (**D**) 600 nm; (**E**) 820 nm; (**F**) 1300 nm. The insets in SEM images are the digital photographs.

Here, in this part, we fabricated monolayer colloidal crystals array using PSCP with diameter of 160 nm, 300 nm, 430 nm, 600 nm, 820 nm and 1300 nm. As shown in Fig. 2, the high quality monolayer PSCP array can be observed. Figure 2A–F show the high order PSCP arrays prepared by 160 nm, 300 nm, 430 nm, 600 nm, 820 nm and 1300 nm, respectively. And the corresponding inset images are the digital photograph of monolayer PSCP array. The high quality of the monolayer PSCP array can be judged by naked eyes. The uniformly color that extends to centimeter dimensions can be observed for all of the samples. The color of the colloidal crystals derives from bragg diffraction of microscopically ordered structures.

**Figure 3:**
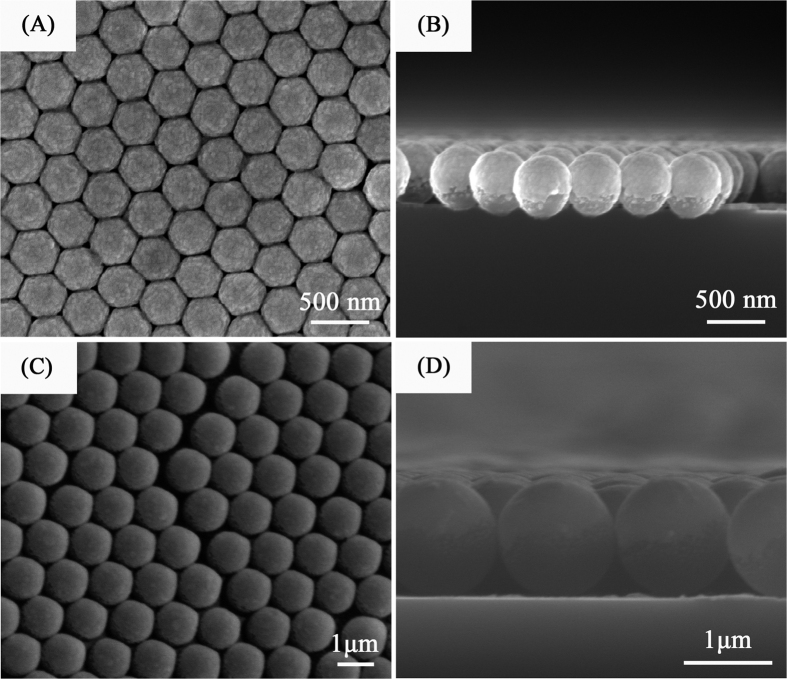
The top-view and side-view SEM images of Ag semishells (40 nm) on different diameter monolayer colloidal crystals: (**A**,**B**) 430 nm; (**C**,**D**) 1300 nm.

The morphologies of AgSS arrays were observed by a field emission scanning electron microscope (FE-SEM). Figure 3 displays the typical SEM images of AgSS@ PSCP arrays. It was clearly observed that most spheres were in contact with six neighboring ones, namely, arranged in a hexagonal close packed structure. Such an arrangement is virtually the same as that for a PS sphere array, indicating that deposition of the Ag layer did not disorder the hexagonal structure of the 2D PS colloidal crystal. For both arrays, the formation of smooth Ag films on the surfaces of the upper hemispheres was verified, as shown in the side-view SEM images of Fig. 3(B,D), respectively. In addition, adjacent Ag half-shells were interconnected by Ag deposition. From these observations, the appearance of strong local electric fields at the interstitial regions is expected.

**Figure 4:**
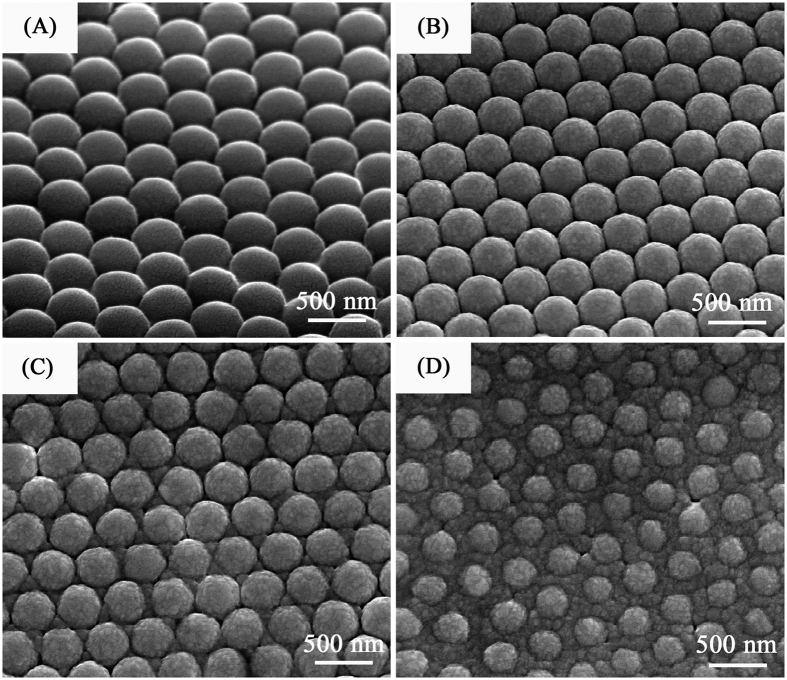
SEM images of AgSS with different Ag film thicknesses. The Ag film thicknesses in (**A**–**D**) are h = 10 nm, h = 40 nm, h = 80 nm, h = 200 nm, respectively. The diameter of the PS microspheres is D = 430 nm.

The morphologies and the SERS activities of the AgSS arrays have been modified by adjusting the PSCP diameter and the Ag film thickness. Figure 4 displays the SEM images of AgSS on 430 nm PSCP monolayer templates array. These SEM images show the roughness on nanoscale, and the nanogaps between AgSS depend on the Ag film thickness strongly. The SEM image in Fig. 4A shows that the Ag AgSS substrate is a perfectly circular dense array when the Ag film thickness is 10 nm. Compared to the PSCP array (as shown the Fig. 1), the morphology of the AgSS substrate has little change under the thin Ag coating film. These small Ag nanoparticles (NPs) are evenly distributed on the surface of PSCP array. When the Ag film thickness gradually increases to 40 nm, the V-shaped gaps between adjacent AgSS gradually decrease and become homogeneous because these small Ag NPs grow up in size and aggregate gradually. At the same time, the nanogap sizes between the adjacent AgSS NPs are gradually decreased, as shown in Fig. 4B. It can be seen that the gap between the AgSS NPs becomes very clear when the thickness of the Ag film increases. As the film thickness increases further to 80 nm (Fig. 4C), the V-shaped gaps begin to coalesce and the surface roughness decreases. As shown the Fig. 4C, compared to 40 nm film (Fig. 4B), the crevices among islands begin to decline and the islands tend to coalesce, which leads to the decreased surface roughness, and a similar result is reported in a previous publication^32^. In addition, due to the mutual connection of adjacent nanoparticles, the nanogap between adjacent nanoparticles almost disappeared. When the thickness of the Ag film further increases to h = 200 nm, as shown the Fig. 4D, the surface roughness is further reduced. Severe distortion deformation of the coated PS microspheres occurs, and the sizes and shapes of the different microspheres apparently become inconsistent. Additionally, the coated microspheres contact each other in some areas, in which some of the gaps were closed and Ag clusters were weakened. Pure Ag film (h = 40 nm) was deposited on the surface of Si substrate, as shown the Fig. S4. The Ag film surface is very smooth, and nanoparticle uniformity is very good. Therefore, in this part, when the Ag film thickness is thin (h = 40 nm), AgSS nanocap arrays form, where lots of “V”-shaped nanogaps exist in adjacent PSCP beads. However, when the Ag film thickness is much larger than 80 nm, these “V”-shaped nanogaps disappear. The Ag nanocaps are connected with each other and the Ag film structures form with “U”-shaped nanogaps between adjacent PSCP beads. The change in the three-dimensional morphology of the nanoscale microsphere arrays would result in a significant influence on the SERS substrate’s hot-spots density and the highly enhanced electromagnetic coupling region, which will significantly affect the Raman-active performance of the substrate.

**Figure 5:**
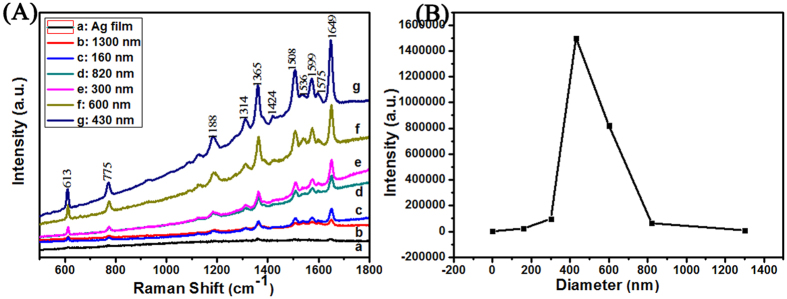
Raman spectra of R6G (1 × 10^−6^ M) are on the AgSS (40 nm) with different diameter monolayer PSCP: (a) 0 nm (Ag film), (b) 1300 nm, (c)160 nm, (d) 820 nm, (e) 300 nm, (f) 600 nm, (g) 430 nm.

The SERS activity of the AgSS (h = 40 nm) was evaluated by using R6G as the probing adsorbate. AgSS (40 nm) at various diameter monolayer monolayer PSCP were used as the SERS samples in order to estimate SERS contribution. The SERS contribution came from the “hot spots” of AgSS nanogaps. Figure 5A displays the SERS spectrum of R6G (1 × 10^−6^ M) with different SERS substrates (AgSS prepared various diameter monolayer PSCP). Here, the SERS excitation source is 514.5 nm. A large number of peaks appear in the spectra curve. These peaks are assigned to the aromatic stretching vibration that at 1365, 1508 and 1649 cm^−1^. Based on our previous researches about the SERS quality for the SERS substrates^33^,^34^, the enhancement factor (EF) for different samples were roughly estimated by comparing the peak intensity at 1649 cm^−1^, as shown Fig. 5B. The enhancement factor (EF) values of R6G in the nanoparticles are calculated the following expression:


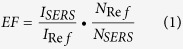


I_SERS_ is the enhanced intensity of the adsorbed R6G molecules on the SERS substrate. The value of I_SERS_ mainly arises from a single molecule layer covering a nanoparticle array, from which other additional molecule layers of analytes on the SERS substrate, as previously reported^34^, do not contribute to Raman gain and can be neglected. I_Ref_ is the spontaneous Raman scattering intensity from the bulk R6G molecules under the laser spot on the blank glass substrate. N_SERS_ is the number of the single-layer molecules covering the SERS substrate under the laser spot. N_Ref_ is the number of the bulk molecules excited by laser on the surface of the regular substrate. In order to obtain the values of these four parameters, we follow the same procedures based on the published literatures^33^,^34^. Using the 50× objective lens, we determine the area of the laser spot size at around 1 μm^2^. We also calculate the area of a single molecule of R6G to be approximately 1 × 10^4 ^nm^2^. Then, the intensity of the peak at 1649 cm^−1^ (EF) is following 3.2 × 10^2^ (Ag film, h = 40 nm), 2.3 × 10^4^ (AgSS, D = 160 nm), 9.8 × 10^4^ (AgSS, D = 300 nm), 1.5 × 10^6^ (AgSS, D = 430 nm), 8.2 × 10^5^ (AgSS, D=600 nm), 6.4 × 10^4^ (AgSS, D = 800 nm) and 9.7 × 10^3^ (AgSS, D = 1300 nm) respectively. Compared with the SERS signals of the Ag film substrate, the R6G’s SERS signals of on the AgSS (D = 430 nm) substrate is about 500 times at the same excitation. Therefore, these AgSS samples have obvious advantages that one is easy to use, another is to provide strong Raman signals. When the R6G probe molecules have adsorbed on AgSS substrates, the intensity of the peak at 1649 cm^−1^ has changed with the different diameter monolayer PSCP, as shown in Fig. 5B. This phenomenon displays that the SERS activity of AgSS substrates could be controlled through different diameter monolayer PSCP. From these spectra curves, we have known that the AgSS substrate prepared by monolayer PSCP (D = 430 nm) has the largest SERS intensity. As shown in Fig. 5, when the diameter of PSCP increased in the AgSS, the intensity of the “hot spots” does not always increase automatically. When the diameter of PSCP continues to increase (from 400 to 13000 nm), the SERS intensity begins to decrease. This reason comes from the different gaps between the adjacent AgSS. It is well known that the “hot spots” can appear at the nanogap region of the dimer when two or more nanostructures contact to each other. And these “hot spots” can induce the strong Raman signals by electromagnetic enhancement mechanism^34^,^35^. The transition dipoles of nanoparticles will produce the plasmonic couple when the nanoparticles close to each other. These plasmonic couple around each particle can provide enhanced electromagnetic fields^36^. The plasmon resonance will shift to red when the distance of gaps region between the adjacent AgSS decreases along the increase in diameter. As a result the enhanced electromagnetic field will strong at the nanogaps region of the nanoparticles. Moreover, when the distance of gaps decreased, the lots of “hot spots” will increase also in order to strong the SERS signals. When the diameter decreases, the morphology of AgSS becomes more planar in order to the morphology of nanoparticles changes from 3D to 2D. The localized field enhancement becomes weaker when the smaller is used on the AgSS substrates. So, when the diameter of PSCP is changed from 400 to 160 nm, the SERS intensity of AsSS decreases.

**Figure 6:**
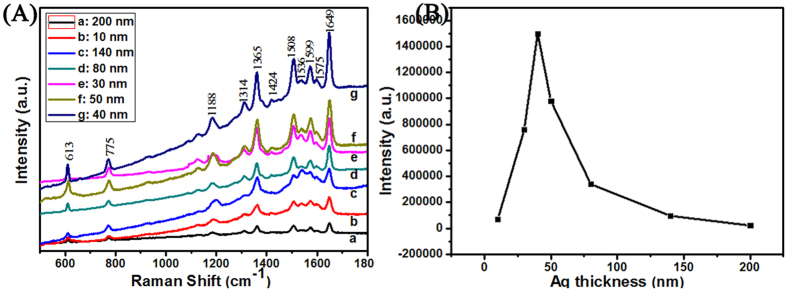
Raman spectra of R6G (1 × 10^−6^ M) are on the AgSS (PSCP = 430 nm) with different Ag film thicknesses: (a) 200 nm, (b) 10 nm, (c)140 nm, (d) 80 nm, (e) 30 nm, (f) 50 nm, (g) 40 nm. The diameter of the PSCP is 430 nm.

Figure 6 displays the Raman spectra of the AgSS substrates (D = 430 nm) with different Ag thicknesses shown in Fig. 4. The results for the SERS performance of the R6G probe molecule (1 × 10^−6^ M) with different Ag coating film thicknesses of 10, 30, 40, 50, 80, 140 and 200 nm are given in Fig. 6A. The enhancement factor (EF) for different samples were roughly estimated by comparing the peak intensity at 1649 cm^−1^, as shown Fig. 6B. Here, the intensity of the peak at 1649 cm^−1^ is following 6.9 × 10^4^ (10 nm), 7.6 × 10^5^ (30 nm), 1.5 × 10^6^ (40 nm), 9.8 × 10^6^ (50 nm), 3.4 × 10^5^ (80 nm), 9.6 × 10^4^ (140 nm) and 2.1 × 10^4^ (200 nm) respectively. After comparison, the AgSS substrate with the 40 nm thick Ag film presents the best R6G probe molecule enhancement results. For the AgSS substrate with a 40 nm thick Ag coating, the main gap range for the V-shaped nanoslits was 10–20 nm, and the density of the 10–20 nm gap nanoslits is larger than that of other substrates with different Ag thicknesses. In this part, the diameter of PSCP is 430 nm, the 10 nm gaps among AgSS were only modulated by the magnetron sputtering coating, which would effectively increase the electromagnetic coupling region and help to enhance the Raman signal for the probe molecule. Moreover, Ag particles and Ag clusters with a clear outline cover the surface of the AgSS with the 40 nm Ag film, which could further enrich the roughness of the three dimensional SERS structure, as shown in Fig. 4. Moreover, to some extent, the multiple scale structures increase the specific surface area of the substrate, which could lead to more probe molecules being adsorbed and increase the field enhancement^37^. As shown the Fig. 4(A–D), more Ag clusters deposited on the PSCP and the V-shaped gap for the 40 nm Ag film than for the other thicknesses, which will contribute to the enhancement in SERS performance. Therefore, the SERS result for the AgSS substrate coated with a 40 nm thick Ag film is better than those for substrates coated with 30 nm and 50 nm thick Ag films. These two points discussed above explain why the AgSS substrate with h = 40 nm provides better SERS signal enhancement than the other AgSS substrates in this work. Figure S5 shows the SERS spectrum of R6G with different concentration that is 10^−7^, 10^−8^, 10^−9^, 10^−10^ and 10^−11 ^M on the on the AgSS substrate (Ag film thicknesses = 40 nm, PSCP = 430 nm). The SERS signal intensities will decrease when the concentration decreases. However, we can still watch the signals at a concentration as low as 10^−11 ^M. Therefore, the AgSS substrate (Ag film thicknesses = 40 nm, PSCP = 430 nm) could be employed as an efficient SERS substrate in this study. Spot-to-spot reproducibility of the signal from the AgSS substrate with h = 40 nm was assessed by Raman mapping wherein spectra were obtained from randomly selected areas. Here, 40 random places were chosen with distances greater than 4 mm from each other. As shown the Fig. S6, the uniformity of the SERS substrate was researched via the mapping for an area of 240 × 240 μm^2^. The impressive maximum relative standard deviation (RSD) value is 2.7%.

**Figure 7:**
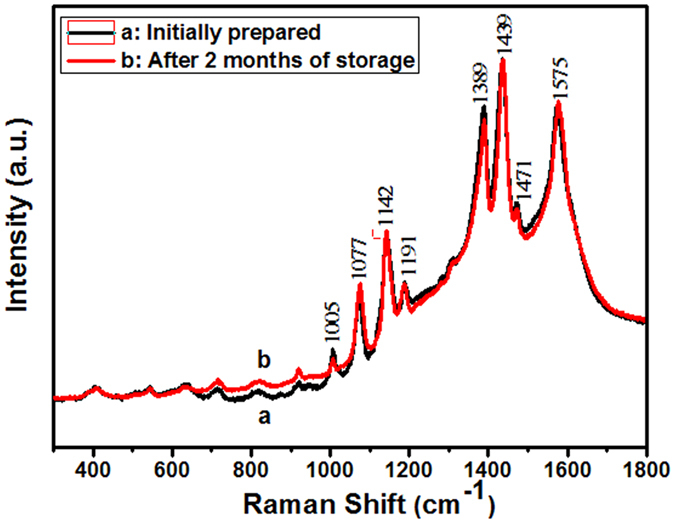
SERS spectra of 4-ATP (1 × 10^−5^ M) are on the AgSS substrates (Ag film thicknesses = 40 nm, PSCP = 430 nm) of initially prepared (a) and after 2 months of storage (b).

Here, we have researched the stability of the AgSS substrates (Ag film thicknesses = 40 nm, PSCP = 430 nm) via measuring the Raman activity with different storage times. The AgSS substrates could give a Raman signal even two months after they were drop coated with 4-ATP molecules. Figure 7 displays the aging of the SERS spectrum for 4-ATP (1 × 10^−5^ M) on AgSS substrates (Ag film thicknesses = 40 nm, PSCP = 430 nm). The dominant peaks at 1575 cm^−1^ (υCC), 1439 cm^−1^ (υCC+δCH), 1389 cm^−1^ (δCH+υCC) and 1142 cm^−1^ (δCH) are ascribed to the b_2_ vibration mode, indicating the charge transfer enhancement is significant^38^. Two other peaks at 1191 cm^−1^ (δCH) and 1077 cm^−1^ (υCC) can be assigned to the a_1_ vibration mode, indicating the electromagnetic enhancement also exists. There is just very a small reduction in the SERS intensity over a period of two months. Therefore, the AgSS substrates display high activity and stability of SERS for detecting 4-ATP molecules.

**Figure 8:**
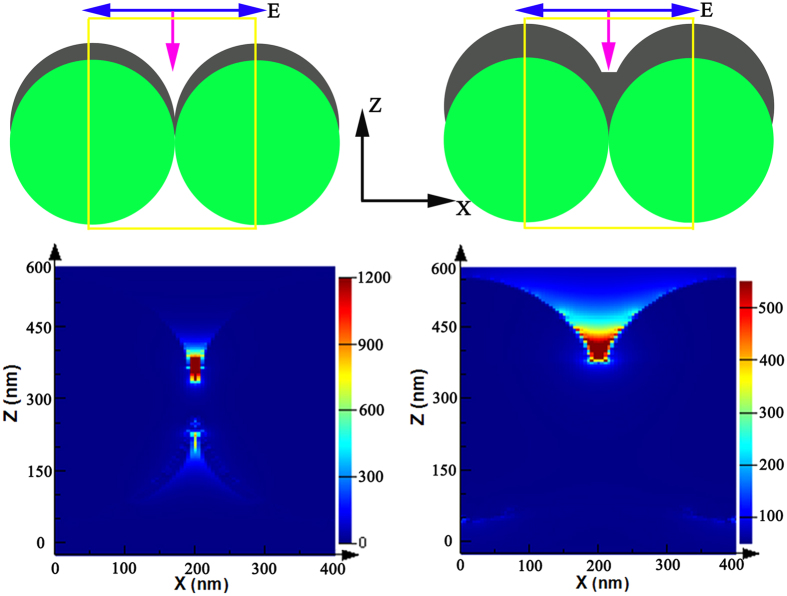
Schematic (**A**,**B**) representation of the simulated structure with AgSS over PSCP. (**C**,**D**) E-field amplitude patterns from the theoretical calculations at the excitation wavelength of 514.5 nm for “V”-shaped gap and “U”-shaped gap.

In this part, we provide by FDTD simulations further observation the possible geometries of highly SERS active sites. We simulate electromagnetic fields distribution on dimers of AgSS on top of PSCP. As shown the Figs 3 and 4, because the AgSS nanogaps are interconnected, we inquire about the “hot-spot” geometry by researching theoretically the effect of the sharpness of the gap between the two AgSS. Figure 8 displays the typical electromagnetic fields distribution for the nanocap array with “V”-shaped and “U”-shaped gaps. Figure 8(A,B) shows two models of AgSS with different shaped gaps: (A) “V”-shaped gap and (B) “U”-shaped gap. Figure 8C displays the enhanced electromagnetic fields distribution that are located at the junction between the AgSS (“V”-shaped gap), which is consistent with the SERS intensity distribution in the previous test. The maximal electric field strength (|E|/|E_0_|)^2^ of 1.25 × 10^3^ can be obtained. For SERS intensity, it is widely believed that |(|E|/|E_0_|)|^4^ is the EF factor for the Raman intensity increases^39^. The maximum Raman enhancement signals for the “V”-shaped gap are 1.5 × 10^6^, which is consistent in agreement with our experimental results (Fig. 6). When the junction between the AgSS is “U”-shaped gap, as shown the Fig. 8D, the maximal electric field strength (|E|/|E_0_|)^2^ of 5.6 × 10^2^. Therefore, we suggest that “V”-shaped gap of AgSS can provide good geometric conditions for electromagnetic fields localization and very large SERS enhancements. Detailed studies of all the geometric parameters influencing the calculations results are beyond the scope of the present work, and will be discussed in detail in the future research.

## Conclusions

In summary, the AgSS ordered arrays were successfully prepared by depositing Ag film onto monolayer PSCP ordered array. The optimization parameters of monolayer PSCP (different diameter) ordered arrays were studied. These AgSS ordered arrays exhibited an ultrasensitive and reproducible SERS property. The SERS properties of AgSS ordered arrays substrates can be controlled through the PSCP diameter and Ag film thickness. When the Ag film thickness is determined (h = 40 nm), AgSS ordered array substrate prepared by 430 nm of PSCP has the largest SERS intensity. The highest enhancement factor of 40 nm thickness AgSS arrays film onto 430 nm PSCP is estimated to be 1.5 × 10^6^. Both the experimental spectra and the calculations of the electric field distribution displayed that the Raman enhancement effect of “V”-shaped gaps is much stronger than that of the “U”-shaped gaps. The study provides a possibility to the facile prepared of SERS substrate with good SERS activity and promising “hot spot” engineering on ordered array surfaces.

### Experimental Details

#### Materials and substrates

Monodisperse PSCP of 160, 300, 430, 600, 820, and 1300 nm in diameter (coefficient of variation of less than 2%) were prepared for the research. The PSCP were made in deionized water solution (different mass fraction, pure colloidal suspensions). 4-aminothiophenol (4-ATP), rhodamine 6G (R6G) and sodium dodecyl sulfonate (SDS) were purchased from Sinopharm Chemical Reagent Co., Ltd. 10 × 10 mm quadrate and 3 inch circular silicon substrates were cleaned following the procedures. Substrates were cleaned with a “piranha solution” for at least 2 h (3:7, 30% hydrogen peroxide/concentrated sulfuric acid). Then the quartz substrates were rinsed extensively with deionized water and dried in a stream of dry nitrogen prior to use.

### Preparation of the monolayer PSCP array

**Figure 9:**
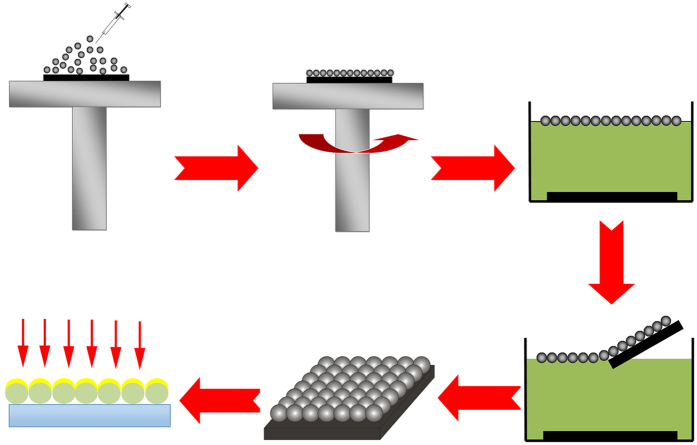
Monolayer colloidal crystals formation form on the surface by the air/water interface selfassembly technology.

To understanding the process of AgSS on PSCP array, the general procedure of self-assembly PSCP array in our case involves six steps, as shown in Fig. 9. Firstly, a drop of aqueous suspension (20 μl) with different concentration of PSCP was deposited on substrate. And next step is drying of a colloidal micropsheres film on the substrate by a spin coater (different spin speed). Unstable submonolayer formed by individually separated colloidal spheres on the substrate. Then, the substrate with PSCP is immersed to the deionized water slowly. The PSCP transform from hydrophilic to hydrophobic since drying of water float and pack into dense at the air/water interface. To consolidate the microspheres, the water surface tension was changed by the addition some of SDS solution and a large monolayer with highly ordered areas was obtained. In the next step, the high ordered formed monolayer can be conveniently picked up by quadrate silicon substrates. Finally, the Ag layer was deposited onto the monolayer PSCP bead arrays.

### Preparation of ordered Ag Semishells array

The AgSS substrates with different Ag film thickness (different thick, as monitored by quartz crystal microbalance) were produced on monolayer PSCP of different sizes in magnetron sputtering (ATC 1800-F, USA AJA). The purity of Ag target is 99.9999%. The working gas was argon and the airflow was 20 sccm. The power of the magnetron was 50 W (voltage of 410 V and current of 100 mA). During deposition, the pressure in the magnetron chamber was 5 × 10^−1 ^Pa and the distance between target and substrate was 10 cm.

### SERS spectra measurements

During SERS spectra measurements, we dropped R6G aqueous solution (10 μL, 1 × 10^−6^ M) on these samples via using an accurate pipet, firstly. Soon afterwards, for getting a uniform molecule membrane about 10 mm^2^, these samples were dried in air at 20 °C. Herein, we provided three same SERS active substrates samples. In addition, for confirming the reproducibility and stability of these samples, we select ten different points on each substrate. We employed Renishaw 2000 model confocal microscopy Raman spectrometer to test SERS spectra. Leica DMLM system was applied for the microscope attachment. Via using a 50× objective, the laser beams were focused onto a spot, and the diameter of the spot is 1 μm. For exciting the SERS, we used the radiation of 514.5 nm. The laser power at the samples’ position was typically 7.2 mW. All of the Raman spectra were recorded in 20 s. At the same time, all of the Raman spectra were recorded by using baseline corrected and noise filtered.

### 3D Optical Simulation

Numerical calculations were completed using the FDTD Solutions program purchased from Lumerical Solutions, Inc. (Vancouver, Canada). The plane wave with 514.5 nm irradiated these samples from the upper side. The periodic boundary conditions were set around the unit cell, and perfectly matched layer (PML) absorbing boundary conditions were used at the top and bottom boundaries of the cell. For ensuring the fields decay completely, the simulation time was 300 fs, and input pulse width was 14.70 fs. The unit cell was designed in foursquare lattices (D * D), where D is the diameter of the nanostructures, as shown in Fig. S7. For simulation, we used a drude dielectric function for Ag^40^ and a refractive index of 1.55 for the underlying PSCP. The refractive index of surrounding medium was 1.0 for air. The dielectric constants of the Si have been taken from Palik^41^.

## Additional Information

**How to cite this article**: Yi, Z. *et al*. Ordered array of Ag semishells on different diameter monolayer polystyrene colloidal crystals: An ultrasensitive and reproducible SERS substrate. *Sci. Rep*. **6**, 32314; doi: 10.1038/srep32314 (2016).

## Supplementary Material

Supplementary Information

## Figures and Tables

**Table 1:** Summary list of the optimization parameters for monolayer PSCP (different diameter) arrays.

PSCP (nm)	PSCP concentration (wt%)	SDS volume (0.01 mol/L, μl)	Spin speed (rad/min)
160	2.4 wt%	600 μl	1600 rad/min
300	3.0 wt%	800 μl	2000 rad/min
430	3.2 wt%	1100 μl	2200 rad/min
600	3.4 wt%	1600 μl	2500 rad/min
820	3.7 wt%	2400 μl	2900 rad/min
1300	4.0 wt%	4000 μl	3600 rad/min
