# Supplementary material for: Ordered array of Ag semishells on different diameter monolayer polystyrene colloidal crystals: An ultrasensitive and reproducible SERS substrate
Source: Sci Rep. 2016 Sep 2;6:32314. doi: 10.1038/srep32314 (PMC5009367; doi:10.1038/srep32314)
Supplement: Supplementary Information [file srep32314-s1.doc]

**Electronic supplementary information**

**Ordered array of Ag semishells on different diameter monolayer polystyrene colloidal crystals: An ultrasensitive and reproducible SERS substrate**

**Zao Yi ^1,2^, Gao Niu^3^, Jiangshan Luo^3^, Xiaoli Kang^3^, Weitang Yao^1,2^, Weibin Zhang^5^, Yougen Yi^4*^, Yong Yi^1,2^**[[1]](#footnote-2)***, Xin Ye^3*^, Tao Duan^1,2*^, Yongjian Tang^1,2^**

*^1^Joint Laboratory for Extreme Conditions Matter Properties, Southwest University of Science and Technology, Mianyang 621900, China*

*^2^Co-Innovation Center for Energetic Materials, Southwest University of Science and Technology, Mianyang 621900, China*

*^3^Research Center of Laser Fusion, China Academy of Engineering Physics, Mianyang 621900, China*

*^4^College of Physics and Electronics, Central South University, Changsha 410083, China*

*^5^ Department of Physics, Dongguk University, Seoul, 100715, Korea*


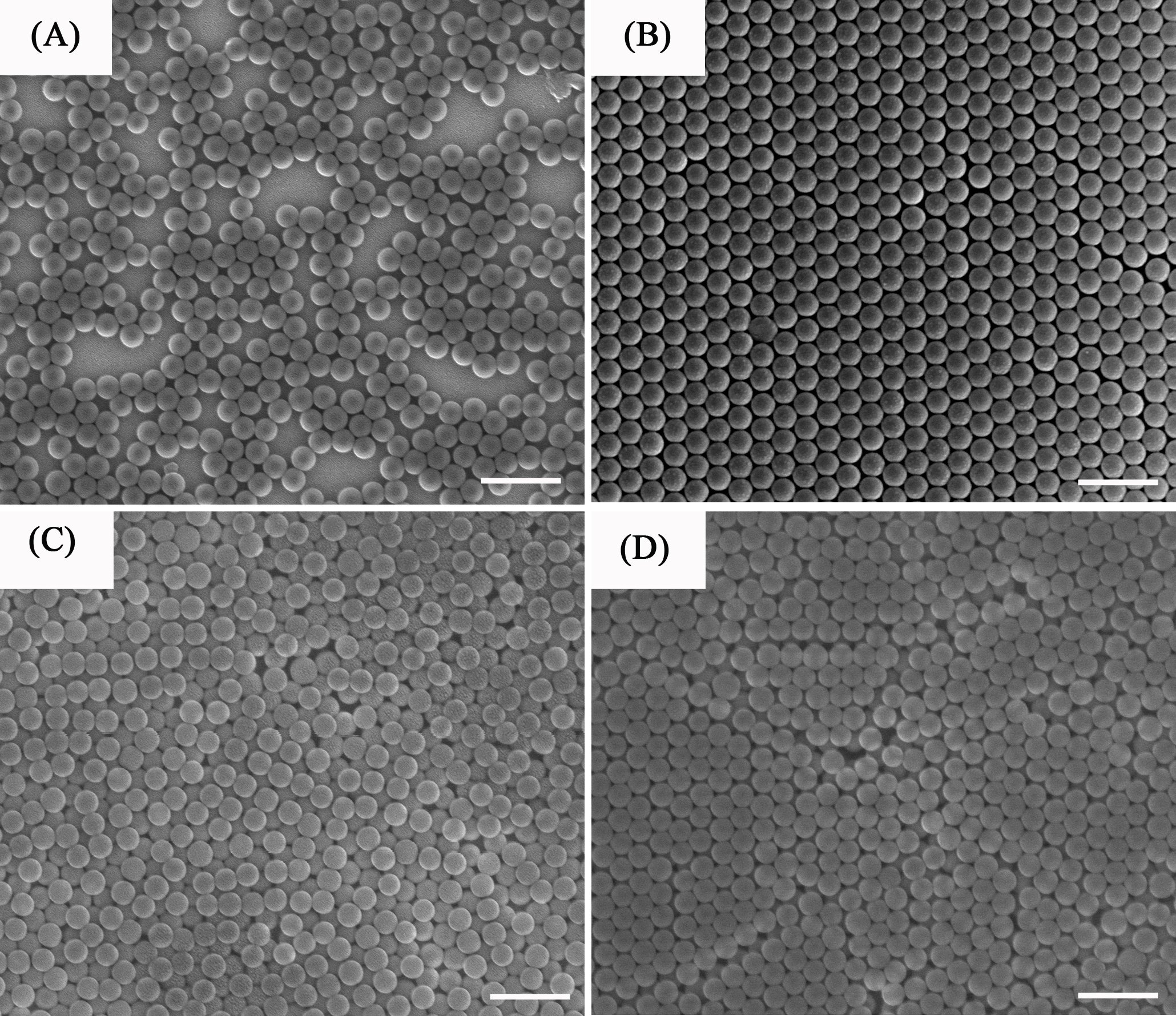


Figure S1: Colloidal crystals were fabricated using PSCP (300 nm) with different concentration: (A) 1 wt%, (B) 3 wt%, (C), 5 wt%, (D), 7 wt%. The volume of SDS (0.01 mol/L) is 800 μl, and the spin speed is 2000 rad/min. (scale bar 1 μm)


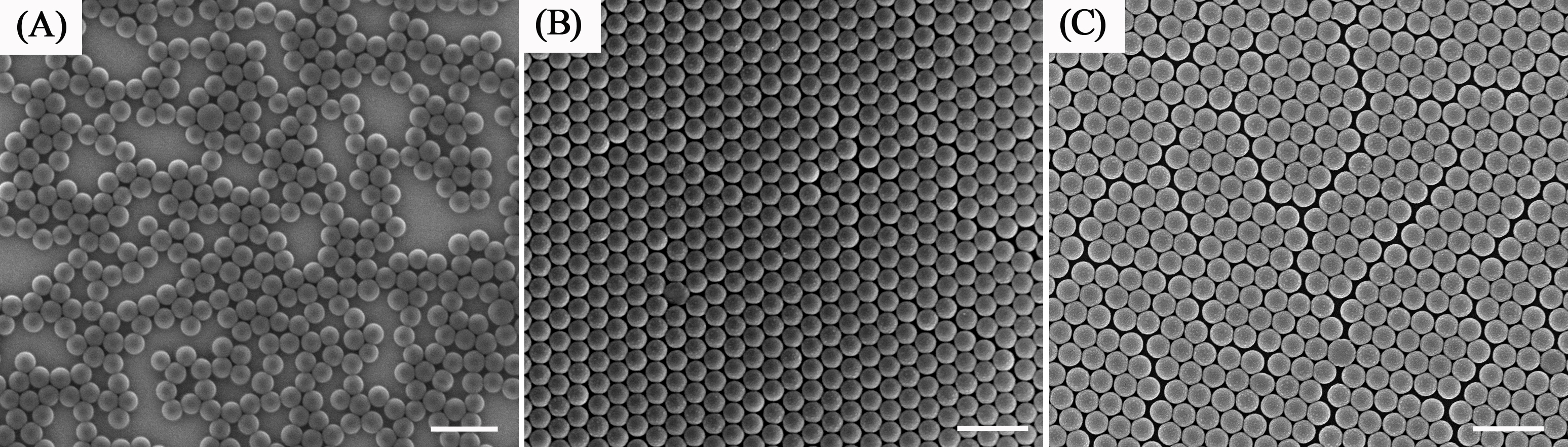


Figure S2: Influence of the SDS volume (0.01 mol/L) on monolayer PSCP (300 nm) formation: (A) 400 μl, (B) 800 μl, (C) 1200 μl. The concentration of PS is 3 wt%, and the spin speed is 2000 rad/min. (scale bar 1 μm)


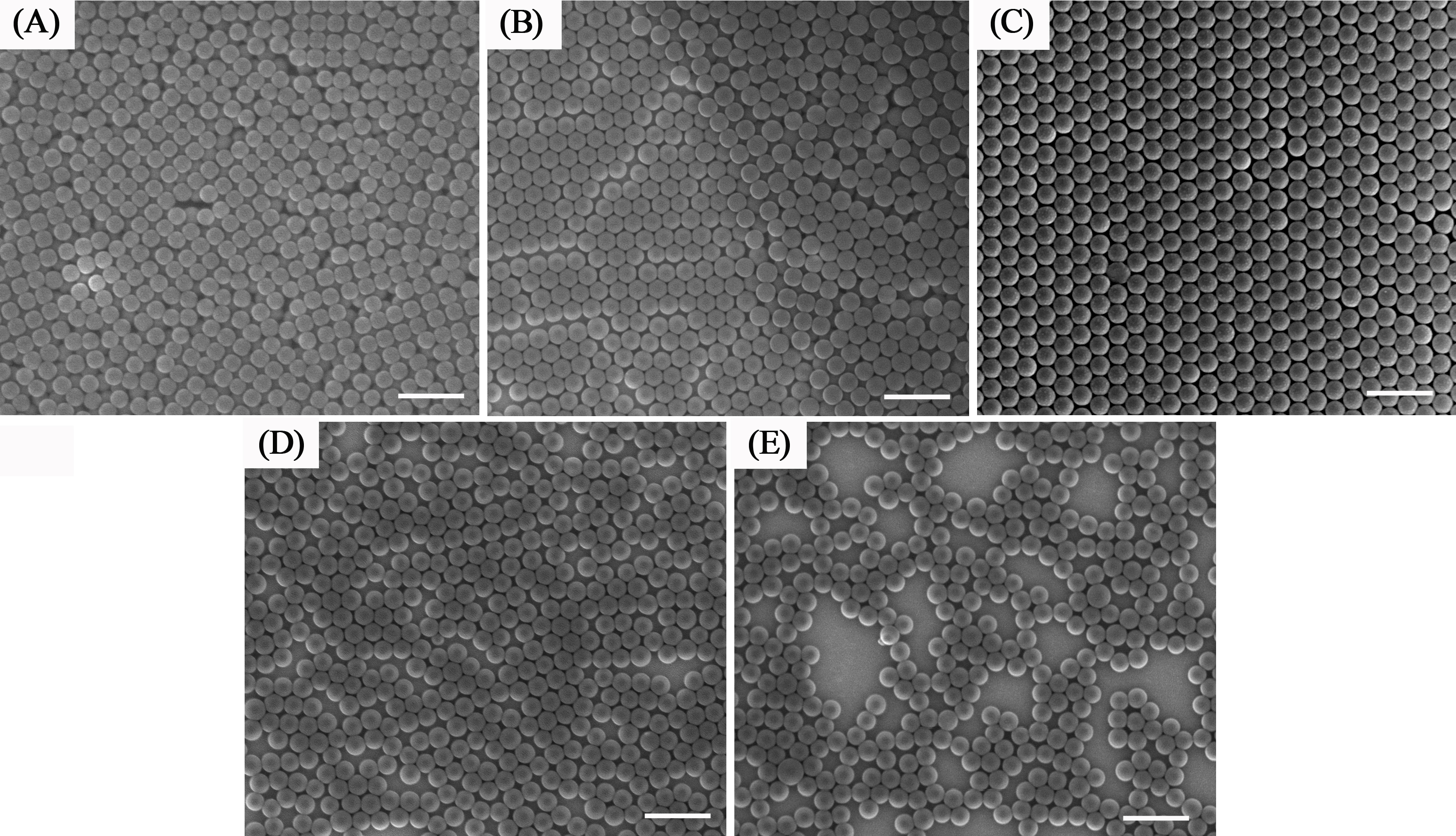


Figure S3: Influence of the spin speed on monolayer PSCP (300 nm) formation: (A) 1000 rad/min, (B) 1500 rad/min, (C) 2000 rad/min, (D) 2500 rad/min, (E) 3000 rad/min. The concentration of PS is 3 wt%, and the volume of SDS (0.01 mol/L) is 800 μl. (scale bar 1 μm)


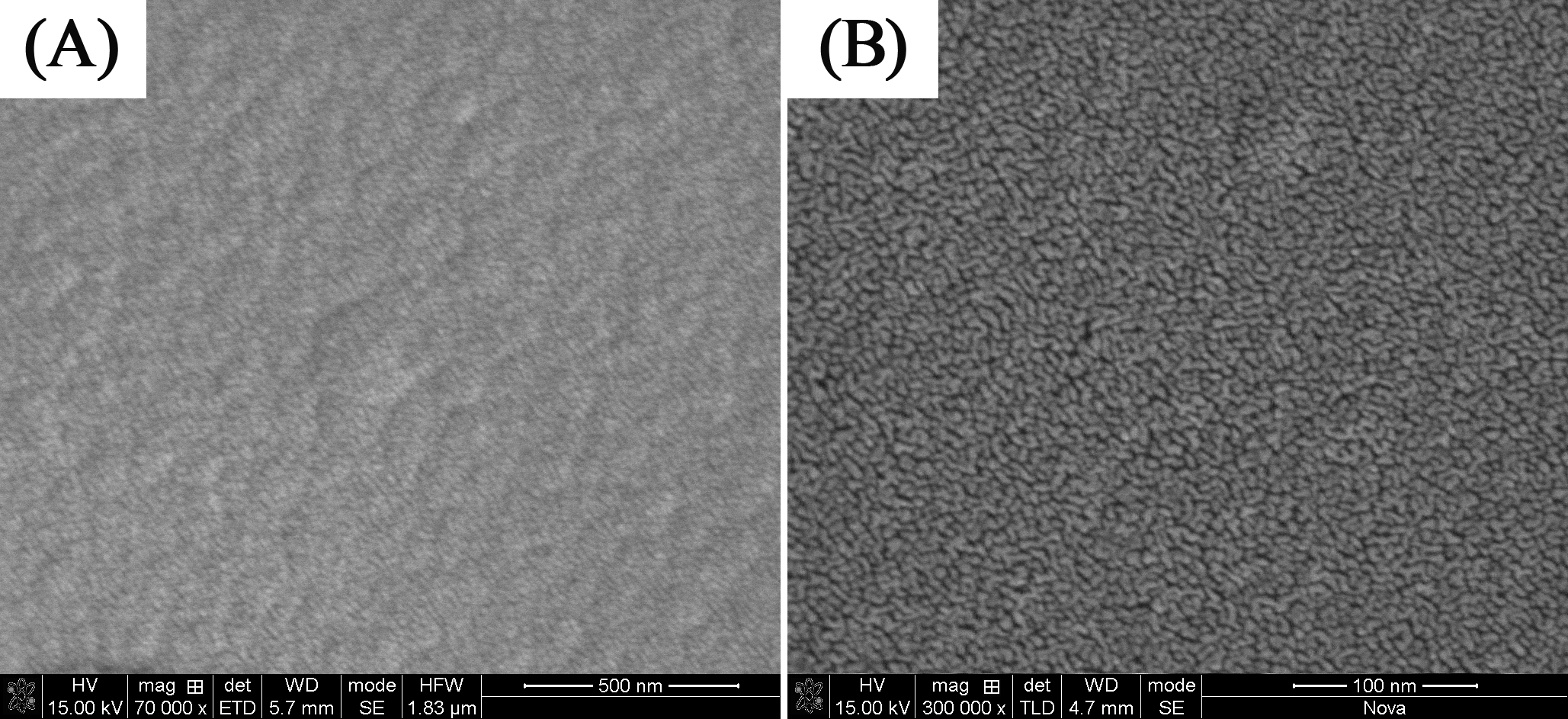


Figure S4: SEM image of the Pure Ag film (h=40 nm) onto the Si wafer.


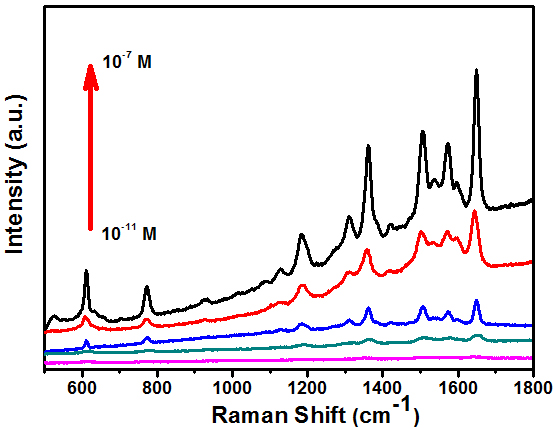


Figure S5: Typical SERS spectra of R6G with different concentrations on the AgSS substrate (Ag film thicknesses=40 nm, PSCP=430 nm).


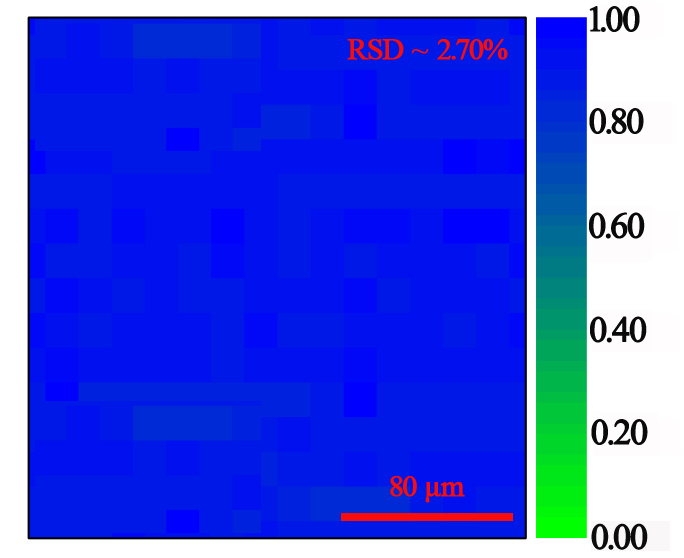


Figure S6: Raman mapping for scanning area of 240 × 240 µm^2^ based on the characteristic R6G Raman band intensity at 1649 cm^-1^. The AgSS substrate’s Ag film thicknesses is 40 nm, PSCP is 430 nm. The intensity of the first point (top-left corner) is normalised as 1.0.


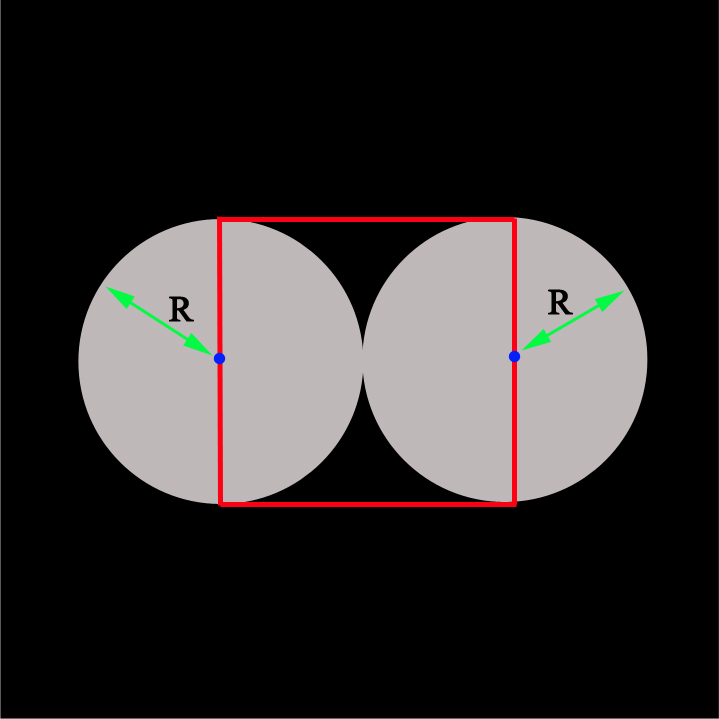


Figure S7: The unit cell of numerical calculation was designed in foursquare lattices (D * D), where D is diameter of nanostructure.

1. ^^ Correspondence should be addressed to Yougen Yi, Yong Yi, Xin Ye and Tao Duan

   Tel: 86-0816-2480830; Fax: 86-0816-2480830

   E-mail address: yougenyi@csu.edu.cn; [myyz1984@csu.edu.cn](mailto:myyz1984@csu.edu.cn); [xyecaep@mail.ustc.edu.cn](mailto:xyecaep@mail.ustc.edu.cn); twcsu2013@csu.edu.cn [↑](#footnote-ref-2)
